# Probability of Transmission of Malaria from Mosquito to Human Is Regulated by Mosquito Parasite Density in Naïve and Vaccinated Hosts

**DOI:** 10.1371/journal.ppat.1006108

**Published:** 2017-01-12

**Authors:** Thomas S. Churcher, Robert E. Sinden, Nick J. Edwards, Ian D. Poulton, Thomas W. Rampling, Patrick M. Brock, Jamie T. Griffin, Leanna M. Upton, Sara E. Zakutansky, Katarzyna A. Sala, Fiona Angrisano, Adrian V. S. Hill, Andrew M. Blagborough

**Affiliations:** 1 MRC Centre for Outbreak Analysis and Modelling, Infectious Disease Epidemiology, Imperial College London, London, United Kingdom; 2 Department of Life Sciences, Imperial College London, South Kensington, London, United Kingdom; 3 The Jenner Institute, University of Oxford, Roosevelt Drive, Oxford, United Kingdom; 4 Institute of Biodiversity Animal Health and Comparative Medicine, College of Medical Veterinary and Life Sciences, University of Glasgow, Glasgow, United Kingdom; Intellectual Ventures Laboratory, UNITED STATES

## Abstract

Over a century since Ronald Ross discovered that malaria is caused by the bite of an infectious mosquito it is still unclear how the number of parasites injected influences disease transmission. Currently it is assumed that all mosquitoes with salivary gland sporozoites are equally infectious irrespective of the number of parasites they harbour, though this has never been rigorously tested. Here we analyse >1000 experimental infections of humans and mice and demonstrate a dose-dependency for probability of infection and the length of the host pre-patent period. Mosquitoes with a higher numbers of sporozoites in their salivary glands following blood-feeding are more likely to have caused infection (and have done so quicker) than mosquitoes with fewer parasites. A similar dose response for the probability of infection was seen for humans given a pre-erythrocytic vaccine candidate targeting circumsporozoite protein (CSP), and in mice with and without transfusion of anti-CSP antibodies. These interventions prevented infection more efficiently from bites made by mosquitoes with fewer parasites. The importance of parasite number has widespread implications across malariology, ranging from our basic understanding of the parasite, how vaccines are evaluated and the way in which transmission should be measured in the field. It also provides direct evidence for why the only registered malaria vaccine RTS,S was partially effective in recent clinical trials.

## Author Summary

Malaria is transmitted to humans by the bite of an infectious mosquito though it is unclear whether a mosquito with a high number of parasites is more infectious than one with only a few. Here we show that the greater the number of parasites within the salivary gland of the mosquito following blood-feeding the more likely it is to have transmitted the disease. A clear dose-response is seen with highly infected mosquitoes being more likely to have caused infection (and to have done so quicker) than lightly infected mosquitoes. This suggesting that mosquito-based methods for measuring transmission in the field need to be refined as they currently only consider whether a mosquito is infected or not (and not how heavily infected the mosquito is). Novel transmission reducing drugs and vaccines are tested by experimentally infecting people using infectious mosquitoes. This work indicates that it is important to further standardise infectious dose in malaria experimental infections to enable the efficacy of new interventions to be accurately compared. The work also provides direct evidence to suggest that the world’s first licenced malaria vaccine may be partially effective because it fails to provide protection against highly infected mosquitoes.

## Introduction

Mosquito-to-human malaria transmission occurs when sporozoites from the salivary gland of the mosquito are injected into the skin during blood-feeding. Parasites then pass to the liver where they replicate, each sporozoite yielding many thousands of merozoites which go on to cause patent infection. Relatively little is known about the population dynamics of malaria between the bite of the infected mosquito and patency. It is currently assumed that the probability of mosquito-to-human transmission is determined simply by the presence of salivary gland sporozoites and not the total number of parasites. For example malaria transmission intensity and the human force of infection are measured using the entomological inoculation rate (EIR) the average number of infectious mosquito bites per person per year [1]. EIR is calculated by multiplying the human biting rate by the proportion of mosquitoes with salivary gland sporozoites and therefore does not explicitly consider how heavily infected the mosquitoes are.

Controlled human malaria infection (CHMI) trials are increasingly being used to evaluate new vaccines [2] and offer a new way to examine the basic biology of the parasite. Volunteers are deliberately infected through the bite of a blood-feeding mosquito or from the direct syringe injection of titrated cryopreserved sporozoites harvested from laboratory reared mosquitoes [3]. The number of syringe injected sporozoites has been shown to correlate with the probability of infection, though the number of viable/infectious sporozoites inoculated is unknown [3, 4]. Therefore it is unclear whether the dose response is caused by an increased probability of the person being injected by a rare viable sporozoite, or whether it is the higher number of viable sporozoites which increases the chance of infection.

Mosquito-delivered sporozoites more reliably recreate the natural infection process than syringe inoculation [4–6], here the probability of infection increases with the number of infectious bites [7, 8]. However a review of the CHMI trial literature and model-system data indicates that no study has thoroughly examined the impact of the number of sporozoites within those bites. The limited number of studies on the subject have given inconsistent results [5, 9–11], potentially resulting from small sample sizes and the use of insensitive statistical methods [12]. Many highly infected mosquitoes fail to inject sporozoites during blood feeding [13–15] so it is again unclear whether the dose response is caused by more bites having a higher probability of injecting a viable sporozoite, or whether the increased quantity of sporozoites injected increases transmission. Currently it is not possible to accurately determine the salivary gland sporozoite burden prior to blood feeding nor the quantity inoculated into the host, which current estimates suggest may vary by multiple orders of magnitude [14–16]. Consequently CHMI studies estimate parasite challenge by dissecting mosquitoes after blood-feeding, counting the number of sporozoites remaining in the salivary glands using microscopy (here referred to as the number of residual-sporozoites, which is scored on a logarithmic scale) [9]. Residual-sporozoite score has been shown to correlate with the number of sporozoites injected into the skin [15].

Time to detectable blood-stage infection is used in CHMI studies to differentiate the efficacy of vaccines which did not elicit sterilising immunity, with longer pre-patent periods indicating a smaller number of parasites passing from the liver to the blood and therefore higher intervention efficacy. Historically, shorter pre-patent periods have been correlated with volunteers receiving more infectious bites [8, 17, 18] but recent analyses of the *Plasmodium vivax* malaria datasets using more appropriate statistical techniques suggest evidence for such an association is weak [12], though once again the impact of sporozoite intensity was not thoroughly investigated.

The relationship between pathogen dose and disease transmission may have important implications for the epidemiology of the disease, how transmission is measured in the field, and the impact of vaccination [19]. It appears intuitive that transmission will increase with the size of the inoculum though there is little empirical evidence to support this assumption and no direct evidence from human malaria. The dose required to infect a host varies dramatically between diseases [20] so the importance of sporozoite load and its relationship with the probability of transmission needs to be clarified.

This study combines results from novel and previously published experimental infections and analyses them with new statistical methods to rigorously determine the impact of the number of residual-sporozoites on the probability of infection. In the CHMI trials analysed here volunteers are bitten by infected mosquitoes until they have received 5 bites from mosquitoes with >10 residual-sporozoites [21]. This ensures that all control volunteers develop malaria [22] though precludes these volunteers being used to investigate factors influencing the probability of naïve human infection. We therefore analyse the results of a recent CHMI study where volunteers were given a partially effective pre-erythrocytic vaccine (PEV) which targets the circumsporozoite protein (CSP) [23]. The analysis is extended to the *Plasmodium berghei–A*. *stephensi* mouse model system [24] where the importance of the number of sporozoites in a mosquito bite can be assessed more thoroughly, with or without the presence of an anti-CSP antibody. To investigate whether sporozoite load influences the size of the liver-to-blood inocula in hosts which become infected (and not just whether or not blood-stage infection develops) the relationship between residual-sporozoite number and the time to patency is assessed. This allows the impact of parasite load to be investigated in a larger human dataset with and without vaccine where most volunteers became infected (S1 Table). For the sake of brevity both humans pre-administered a pre-erythrocytic vaccine and mice pre-administered the anti-CSP 3D11 antibody are referred to as being given a PEV, whilst those that did not are called naïve.

## Results

**Fig 1:**
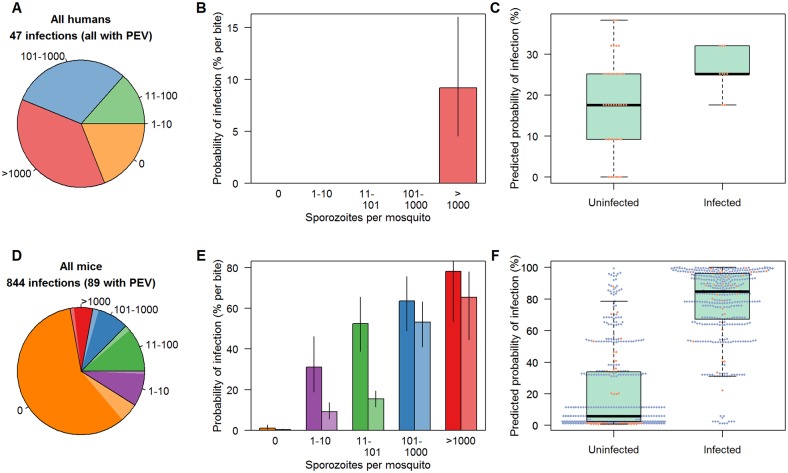
The association between the number of salivary gland sporozoites and the probability of infection in humans (A,B,C) and mice (D,E,F). In panels A,B,D,E naïve infections are denoted by a darker shade of colour whilst hosts given an anti-circumsporozoite protein vaccine or antibody (Pre-Erythrocytic Vaccine -PEV) are a lighter shade. Unvaccinated control humans are excluded from the analysis in (A-C) as all received very high levels of parasite challenge and all developed infection (so there is no variability to test the association with residual-sporozoite load). Panels (A) and (D) show the number of sporozoites in the salivary glands following blood-feeding used in the different experimental infections (be it 0 (orange), 1–10 (purple), 11–100 (green), 101–1000 (blue) or >1000 (red). Panels (B) and (E) show model predictions of the probability that a single bite from a mosquito with a given number of salivary gland sporozoites following blood-feeding will induce patent infection (black lines show 95% confidence interval estimates on model predictions). Panels (C) and (F) show model predictions for overall probability of infection for each individual human and mouse and as estimated using the best fit model. Each point gives the prediction for an individual volunteer according to the number of bites they receive, the residual-sporozoite score of those individual bites (taken from panel (B) and (E) and whether they received a PEV (orange dots) or were untreated (blue dots).

The association between the number of residual-sporozoites and the probability of infection was assessed in 47 vaccinated volunteers (challenged with *Plasmodium falciparum* using *Anopheles stephensi* mosquitoes), 9 of which became infected (Fig 1A). Though there was no difference in the mean (or total) residual-sporozoite number in mosquitoes biting persons who became infected or remained uninfected (S2 Table) a binomial model [25] shows that infection was significantly more likely from mosquitoes with >1000 residual-sporozoites (Fig 1B). The best fit model indicates that mosquitoes harbouring >1000 sporozoites had a per bite transmission probability of 9.2% (4.5%-16.0%) whilst those with a lower number of parasites did not measurably contribute to transmission (Fig 1B, see S3 Table for the results of the full model comparison). This model is used to estimate the probability that a volunteer became infected according to the number of residual-sporozoites in the bites they received (Fig 1C). Though there is still considerable variation, the predicted probability of infection is significantly higher in infected volunteers (*p* = 0.040) supporting the original analysis that mosquito parasite load influences the (per bite) probability of mosquito-to-human transmission. None of the 13 volunteers who received 0 or 1 bites from mosquitoes with >1000 residual-sporozoites became infected.

The analysis is extended to the mouse model system [24] where the importance of the number of sporozoites in a mosquito bite can be assessed more thoroughly, with or without the presence of PEV. A total of 844 mice were included in the analysis, 99 of whom were given a PEV (Fig 1D). Results show a clear dose-dependency, with mosquitoes with a higher number of residual-sporozoites being more likely to transmit malaria both in the naïve mice, and those given a PEV. The probability of infection in a naïve host from a single bite is 32% (19%-46%) from mosquitoes with 1–10 sporozoites and 78% (53%-93%) from those with >1000 sporozoites (Fig 1E). The approximately linear increase in transmission probability with the log-sporozoite number suggests that the per-sporozoite transmission probability is a negative density-dependent process. Model predictions of the probability of infection for each mouse (according to the number of residual-sporozoites of the bites they received) are compared to whether or not they became infected (Fig 1F). There is a significant association between infection status and the model-derived probability of infection (*p*<0.0001). The model is able to predict infection relatively accurately (for example 95.4% of mice with a >95% probability of infection became infected). Greater variability is seen in the probability of infection for mice than for humans. This is because in the rodent dataset the number of bites a mouse receives varies (in addition to the number of residual-sporozoites within those bites) whereas in the human dataset all volunteers receive 5 bites from mosquitoes with >10 residual-sporozoites.

Mosquitoes with no detected residual-sporozoites by microscopy can, very rarely, infect mice. In total, 291 naïve mice were bitten by mosquitoes with no detectable salivary gland sporozoites following blood-feeding. Of these 13 (4.5%) went on to develop a blood stage infection. The lack of observable sporozoites could be caused by mosquitoes injecting all sporozoites during blood-feeding, or due to measurement error in the counting process. If all mice bitten exclusively by mosquitoes with no detectable sporozoites were removed from the analysis then each additional bite from mosquitoes with no detected residual-sporozoites reduces the probability of infection by 4.6% (0.5%-7.4%). Comparing this model to a reduced version where zero scores have no contribution to transmission shows that including the negative impact of these bites does significantly improve model fit (Akaike information criterion, AIC, with 0 = 445.9, AIC without 0 = 452.4). The cause of this negative impact on transmission is unknown and requires further investigation though it could result from a non-specific immune response initiated by an uninfected mosquito bite[26]. As expected the PEV significantly reduces the probability of infection, reducing the per bite transmission probability by 29.1%. The most parsimonious model indicates that the PEV is more effective against lower sporozoite challenges. It reduced the probability of infection by 70.5% for bites with <101 residual-sporozoites and 16.3% in mice bitten by mosquitoes with >100 sporozoites (AIC constant efficacy model = 623.5, variable efficacy model = 622.5, Fig 1E).

**Fig 2:**
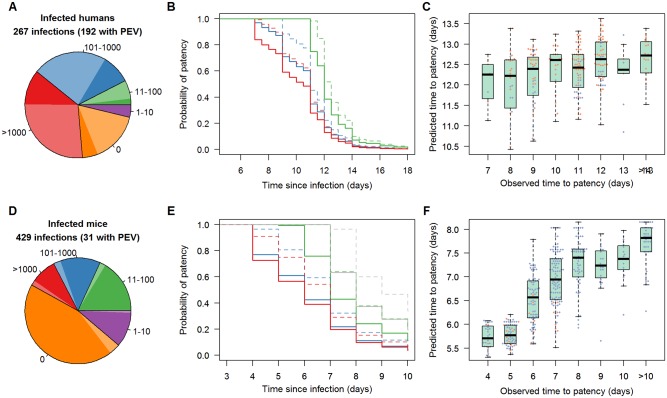
The association between the number of salivary gland sporozoites and the duration of the pre-patent period in humans (A,B,C) and mice (D,E,F). In panels A,B,D,E unvaccinated/untreated control infections are denoted by a solid line (and darker shade of colour) whilst humans given a pre-erythrocytic vaccine (PEV) candidate or mice given an anti-circumsporozoite protein antibody (PEV) are shown by a dashed line (lighter shade of colour). Panels (A) and (D) show the number of sporozoites in the salivary glands following blood-feeding used in the different experimental infections. Panels (B) and (E) show the survival curves predicted by the model for the time between sporozoite inoculation and patent parasitaemia. Grey, green, blue and red lines shows predictions for a vertebrate host with 5 bites from mosquitoes with 1–10, 11–100, 101–1000 and >1000 salivary gland sporozoites after blood-feeding, respectively (grey line is omitted from B as no human volunteers received bites solely from mosquitoes with 1–10 residual-sporozoites). Panels (C) and (F) show the ability of the best fit model to predict the time to patency for humans and mice. Each point gives the prediction for an individual volunteer according to the residual-sporozoite score of biting mosquitoes and whether they received a PEV (orange dots) or were untreated (blue dots).

The number of residual-sporozoites significantly influences the time to patency in both naïve volunteers and those given different PEVs. Data from a total of 267 CHMI volunteers who developed malaria were analysed, 192 of these had received a PEV candidate (Fig 2A and 2B). The best fit survival analysis model was unable to differentiate between mosquitoes with no detectable salivary gland sporozoites and those with 1–10 post-feeding perhaps because the numbers of bites with 1–10 sporozoites was relatively low. A full description of the time invariant components of the survival analysis are given in S4 Table. Importantly, the model predicts that naïve volunteers who were infected by 5 mosquitoes with >1000 sporozoites would have detectable parasitemia on average >2 days earlier than those infected by 5 mosquitoes with 11–101 sporozoites. There is a significant association between observed time to patency and survival analysis model predictions based on the number of residual-sporozoites (Fig 2C, *p*<0.0001). Time to patency analysis was repeated with those *P*. *berghei* challenges of mice which developed infection (Fig 2D–2F). Data from 429 mouse experimental challenges that generated blood-stage infection were used in the survival analysis, 31 of which had received a PEV. These results are consistent with the human data, with naïve mice and those given PEV reaching patent parasitemia earlier if they were bitten by mosquitoes with higher number of residual-sporozoites. Again there is a significant association between observed time to patency in mice and model predictions (Fig 2F, *p*<0.0001). In both the human and rodent system the number of bites and the associated residual-sporozoite loads explains some of the variability in time to patency but not all. This is why the variation seen in observed time to patency is substantially greater than that predicted by the model. The causes of this additional variation are unknown though in the human dataset it could be due in part to the different efficacies of the PEV candidates administered. There is greater variability in the predicted time to patency in the rodent system as the number of times the mice were bitten had a bigger range (between 1 to 10 bites) than the human dataset where all volunteers were bitten by 5 mosquitoes with >10 residual-sporozoites.

**Fig 3:**
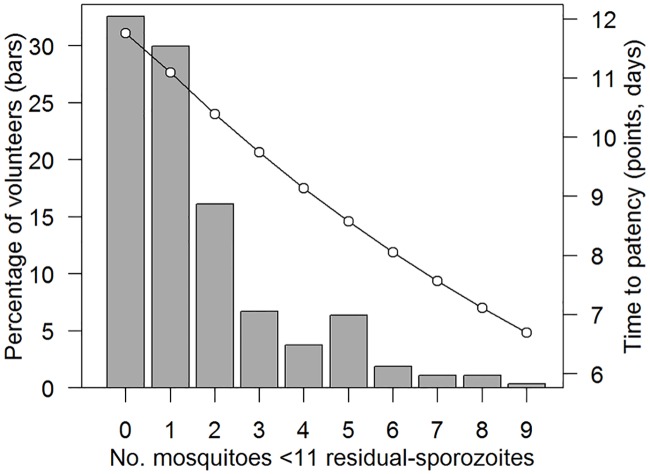
The frequency and impact on the time to patency in humans of mosquito bites with ≤10 residual-sporozoites. Grey bars show how many volunteers received bites from mosquitoes with ≤10 salivary gland sporozoites. Black dots (and line) show model predictions for the mean time to patency for volunteers with different number of (≤10) bites. As the impact of the lightly infected bites will depend on the number of residual-sporozoites in the bites the volunteer received, in this example all volunteer receive 5 bites from mosquitoes with 101–1000 residual-sporozoites in addition to the extra mosquito bites with ≤10 residual-sporozoites.

Human volunteers bitten by ‘additional’ mosquitoes that had ≤10 residual-sporozoites (in addition to the other 5 bites of >10 residual-sporozoites) had a shorter time to patency (Fig 3). These lightly infected mosquitoes are currently not considered infectious in CHMI trials though they appear to have a significant impact on transmission (S4 Table). Similar results were seen in the mouse data, with each additional bite with ≤10 residual-sporozoites reduced the time to patency in infected mice. In time-to-patency data of both humans and mice the best fit model did not differentiate between bites from mosquitoes with no detectable and 1–10 residual-sporozoites. The occurrence of mosquito bites with ≤10 sporozoites were relatively common in CHMI trials. On average each infected volunteer received 1.6 bites from mosquitoes with ≤10 residual-sporozoites, with 2/3 of the volunteers receiving one or more (Fig 3). The impact of these additional bites depends on the other bites that the volunteer received. For example, if a volunteer was bitten by 5 mosquitoes with 101–1000 salivary gland sporozoites then the additional bites reduced the time-to-patency by on average 1.5 days (Fig 3).

## Discussion

Together the human and rodent data provide strong evidence that mosquitoes with more salivary gland sporozoites post-feeding are more infectious. Though this cannot currently be tested directly *in vivo*, the most plausible explanation is that that mosquitoes with a higher number of residual-sporozoites injected more sporozoites into the skin during blood-feeding which increased the probability and the speed of the ensuing blood-stage infection. Whilst the probability of infection analysis could only be tested in humans who had been given a PEV, the consistent association between residual-sporozoites and the time to patency in naïve and vaccinated volunteers and the mouse experiments suggest that parasite intensity, and not just the number of infectious bites, is key to understanding the underlying biology of malaria transmission. The mouse experiments indicate a continuum of infection: with every sporozoite injected into the vertebrate increasing transmission.

The relationship between the number of parasites in the salivary gland and the size of the inoculum is poorly understood. Early laboratory work proffer apparently contradictory results [9, 13, 16, 27]. The thin diameter of the mosquito proximal duct means that only one or two sporozoites can pass down it at the same time [13] which supported the hypothesis that inoculum size, if confined to the void volume of the salivary duct, would be independent of sporozoite gland burden. Nevertheless more recent experiments which better resemble natural transmission show a clear positive association between residual-sporozoite load and the number of sporozoites injected into mice [15]. On blood feeding the majority of sporozoites appear to be injected during the first few seconds [13, 27] suggesting their presence in the duct prior to the bite being taken. The number in the ducts will be dependent upon the density of sporozoites in the glands, the time over which they have accrued in the duct since last feeding (on blood or sugar) and the available volume in the duct. There could be additional reasons other than the number of parasites injected which could explain why heavily infected mosquitoes are more infectious. These could include increased (per sporozoite) infectivity [28] or more frequent mosquito probing [29] and further work will be required to determine the causes of the observed infectivity.

Previous studies that failed to find a relationship between residual-sporozoite number and infection used relatively insensitive statistical methods [5, 11] which may have caused them to be underpowered to detect a difference due to the wide variability in the size of the inoculum from similarly infected mosquitoes. The linear increase in the probability of infection on the logarithmic scale (Fig 1E) shows that for every additional residual-sporozoite the increase in transmission declines (i.e. mosquito-mouse transmission is a negative density-dependent process). This has been observed by others [13] and could be modulated by the limited diameter of the duct restricting passage of sporozoites in highly infected mosquitoes. The linear increase in the probability of infection on the logarithmic scale (Fig 1E) shows that for every additional residual-sporozoite the increase in transmission declines (i.e. mosquito-mouse transmission is a negative density-dependent process). This has been observed by others [13] and could be due to the limited diameter of the duct restricting passage of sporozoites in highly infected mosquitoes.

This analysis allows vaccine efficacy estimates to be standardized from mosquito delivered CHMI trials. It is very technically difficult to homogenize the number of salivary gland sporozoites within a mosquito before blood-feeding, making it hard to fully control the infection challenge from CHMI mosquitoes. Including sporozoite intensity information in the analysis of CHMI studies could help reduce between volunteer variability (lowering sample sizes) [21] and improving the characterisation of immune responses [30]. The number of volunteers used in CHMI trials is understandably small. This study shows that failing to account for the (random) variability in parasite challenge will make PEV candidates harder to compare, both within and between studies. The results from the human and murine systems are consistent. In the rodent model a naïve mouse would require 2 bites from mosquitoes with >1000 residual-sporozoites or 4 bites from mosquitoes with 11–100 residual-sporozoites to ensure a 95% probability of infection. This is in line with the human data where all naïve volunteers became infected having received 5 bites from mosquitoes with >10 sporozoites. Naïve humans could not be included in the analysis as the parasite challenge was so high that all became infected (so there was no variability in that dependent variable). Nevertheless, the consistent pattern seen in mice with and without PEV suggests that residual-sporozoite number would have been associated with the probability of infection in naïve volunteers had parasite challenge been lower. By comparison with published estimates on the infectivity of *P*. *berghei* sporozoites [31], the observed probability of infection is high suggesting current arrangements for laboratory transmission have increased overall transmission probability.

The epidemiological importance of parasite intensity will depend on the distribution of sporozoites in wild mosquitoes and whether similar trends are observed in people with a prior history of malaria infection. All the volunteers analysed here were malaria naïve and received all infections within minutes of each other. It will therefore be important to repeat the experiments on people from malaria endemic regions who may have acquired a degree of immunity to infection. It will also be necessary to confirm the dose effect in singly bitten humans as it is unlikely that many people in malaria endemic Africa will be bitten by 5 infectious mosquitoes in such a short time period. There are relatively little data on the number of sporozoites in naturally infected mosquitoes [32–35] though laboratory reared and infected *A*. *stephensi* have been suggested to inject a similar number of sporozoites to natural infections [9]. In a very low transmission site on the Thai–Myanmar border a recent study reported a geometric mean of 57 sporozoites per mosquito (range 9–11,428) [33]. This compares to earlier studies in Africa and PNG where endemicity was higher and geometric means were >4000 (range 150–10,000) [32, 34]. In these datasets the distribution of sporozoites between mosquitoes appears highly over dispersed (aggregated), with a large number of lightly infected mosquitoes and few with very heavy infections. Notwithstanding the above, more than 45% of infectious mosquitoes caught in a low transmission site in Kenya had >1000 salivary gland sporozoites [35], the same number of residual-sporozoites that was associated with successful infection in the anti-CSP CHMI.

In this study mice were given anti-CSP antibody by passive transfer (*i*.*v*), whilst humans were vaccinated with the anti-CSP RTS,S/AS01B. RTS,S is thought to induce T cell responses as well as antibody. Though we see a similar relationship between both anti-CSP interventions disentangling the impact of the different immune responses is beyond the scope of this study. In both the human and mouse data analysed here the anti-CSP interventions were more effective against bites from mosquitoes with a lower residual-sporozoite number. This may explain why recent Phase II and III trials of RTS,S/AS01 (which targets CSP) were partially effective as the vaccine may only be providing sterilizing immunity against lightly infected mosquitoes [36]. Further work is needed to establish whether the anti-CSP antibodies/immunity provided sterilizing immunity against all bites from mosquitoes with a low number of residual-sporozoites (a “threshold” type of protection) or whether it prevents infection from a certain percentage of inoculated sporozoites (a “leaky” type of vaccine (see S1 Fig for a graphical explanation of the two hypotheses). The RTS,S/AS01 Phase III trial was not powered to detect a difference between sites but evidence suggests that it had a higher efficacy against clinical malaria in low transmission settings [37]. Though this is likely to be caused in part by different levels of immunity in the human population variable parasite challenge could also have contributed. It is unknown whether mosquito parasite challenge will diminish as the disease is successfully controlled (and therefore vaccine efficacy might be expected to rise) or whether the sporozoite dose may remain broadly the same [38].

The work shows that lightly infected mosquitoes (with ≤10 residual-sporozoites) contribute to transmission though they have a lower chance of causing onwards infection than more heavily infected mosquitoes. In areas approaching local elimination a large proportion of mosquitoes are thought to be infected by people with low density infections [39]. The contribution of different infection classes to this reservoir of infection has been assessed by their ability to infect mosquitoes [39] (human-mosquito-transmission) but has failed to consider how onwardly-infectious these mosquitoes might be (mosquito-to-human transmission). Work presented here strongly suggests that not all mosquitoes are equally infectious and therefore low density human infections might have a smaller epidemiological impact if they result in less infectious lightly infected mosquitoes [33]. Further work is needed to investigate this hypothesis as it will have consequences for the required sensitivity of malaria diagnostics and the effectiveness of different drug-based control strategies [40].

Malaria transmission intensity in the field is currently measured using estimates of the number of infectious mosquito bites per person per year (the entomological inoculation rate, EIR) [1]. This metric does not explicitly distinguish between light and heavy infections and therefore fails to describe accurately the onwards infectivity of mosquitoes. This will reduce its sensitivity and potentially generate additional bias in different settings [1]. The proportion of infectious mosquitoes in EIR estimates is typically measured by ELISA which is relatively poor at detecting infection in mosquitoes with fewer than 150 sporozoites [41]. The work presented here shows that these infections contribute to transmission thus causing the human force of infection to be underestimated (particularly in areas approaching local elimination [33]). The EIR is central to our understanding of malaria and how control interventions are compared. The influence of the number of parasites on mosquito-to-human transmission could therefore have wide reaching implications across malariology.

## Materials and Methods

### Ethics Statement

All human data analysed here has previously been published elsewhere. See individual publications for specific ethical statements. All animal procedures were performed in accordance with the terms of the UK Animals (Scientific Procedures) Act (PPL 70/7877) and were approved by the Imperial College Animal Welfare and Ethical Review Body (AWERB) LASA guidelines were adhered to at all points. The Office of Laboratory Animal Welfare Assurance for Imperial College covers all Public Health Service supported activities involving live vertebrates in the US (no. A5634-01). Recovery anaesthesia and terminal anaesthesia was performed during the course of this study as per PPL 70/7877. Anaesthesia used was Ketamine/Rompun, and Schedule 1 was performed by overdose, exsanguination or cervical dislocation as part of Home Office approved regulated procedures.”

### Experimental Design

A full outline of the volunteer selection, procedure and ethical considerations of the humans challenge studies is given by Sheehy et al. [42] whilst the mouse data was generated using methods outlined in Blagborough et al. [24]. Briefly laboratory reared *Anopheles stephensi m*osquitoes were fed on *Plasmodium* infected blood and maintained for ~21 days to enable them to develop salivary gland sporozoites. *P*. *falciparum* NF54/3D7 strain was used to infect humans, *P*. *berghei* clone 2.34 was used in the mouse studies. Mosquitoes were considered to have delivered sporozoites only if they ingested red blood cells in the midgut. Fed mosquitoes were immediately dissected and the number of sporozoites remaining in the salivary glands categorized on a logarithmic scale: 0 (no sporozoites), 1 (1–10), 2 (11–100), 3 (101–1000), 4 (>1000) [9]. In the human trials the feeding procedure was repeated until each volunteer had received 5 bites with >10 residual-sporozoites (in studies carried out before 2004 this value was of score 3 or above, S1 Table). Mice were bitten 1, 2, 3, 4, 5 or 10 times irrespective of the residual-sporozoite score. All bites were received in a short period of time so it is assumed that all mosquitoes contribute equally to the probability of infection and the time to patency (i.e. bite order is not important). Historical CHMI challenge data were collated by returning to the original paper records of each volunteer. Infections where information on the full parasite challenge was unavailable were excluded from the analysis.

The CHMI trials were originally carried out to test different potential malaria PEV candidates (S1 Table). All human control volunteers developed patent infection so could not be used in the probability of infection analysis. Instead data from a single study where 33 volunteers received a three dose regime of RTS,S/AS01B (18) that provided sterilizing immunity to 14 patients (who were subsequently re-challenged 6 months later). There was no significant difference in the impact of the two intervention arms or between initial challenge and re-challenge infections so all data were pooled making a total of 47 experimental infections.

In the mouse study data was collated from population studies to evaluate the impact of compounds inhibiting malarial transmission from mouse-to-mosquitoes which are not present during mosquito-to-mouse transmission (atovaquone [24], primaquine [43], NITD609 [43], artemether + lumefantrine [43] and OZ439 [43]). Other populations of mice were administered the monoclonal antibody 3D11 which targets the same homologous circumsporozoite protein (PbCSP) as the human vaccine RTS,S (PfCSP) which is the first malaria vaccine to be licensed [44]. A number of the human vaccines presented in S1 Table also target PfCSP. Asexual parasite density was regularly measured to determine infection status and time to patency (every day from day 4 to 10 for the mouse model and twice daily from day 6 to 15 and daily up to day 21 in the human volunteers). Volunteers were given a curative anti-malarial treatment on the detection of blood-stage parasites by microscopy. Up to this time-point samples were analyzed by using quantitative real-time PCR (qPCR, analyzing 150μl blood) [42] whilst parasiteamia in mouse samples were quantified by microscopy (by reading 4 microscopy fields, each with approximately 300 red blood cells) [24].

### Statistical Analysis–Probability of Infection

The mean residual-sporozoite score (the average score across all mosquito bites) and the total residual sporozoite score (the sum of the scores across all bites) were calculated for all hosts. To disentangle the effect of the number of bites and the residual-sporozoite scores within those bites a linear-mixed effect model is used to test whether hosts that got infected were bitten by mosquitoes with a higher residual-sporozoite score. The mean and total residual-sporozoite score is taken as the dependent variable whilst a binary value denoting whether or not the host developed infection is included as a fixed effect. The number of bites received is incorporated as a random effect allowing the difference in mean and total residual-sporozoite scores to vary between biting groups. Models with and without the fixed effect were compared using a likelihood ratio test to see whether there was a significantly different residual-sporozoite scores between infected and uninfected hosts.

It is likely that the relationship between the probability of infection and residual-sporozoite score is non-linear so a more advanced binomial statistical model is tested [25]. It is assumed that all bites are independent, i.e. the transmission probability of a bite from a mosquito with a certain residual-sporozoite score is the same irrespective of the number of other bites received. The overall probability of infection is described by a binomial distribution allowing data from multiply bitten hosts to be accurately included (i.e. the probability of infection from two bites each of which have an infection probability of 50% is 75%, not 100%). Sporozoite score is categorised into bins on the logarithmic scale so the probability of infection is estimated for each group independently (i.e. not using a continuous function) as the distribution of parasite numbers within these bins is unknown. Let *ϕ* denote the probability of a susceptible host becoming infected and *b*_*j*_ be the per bite transmission probability (the proportion of bites by an infected mosquito with a residual-sporozoite score *j* which go onto develop patent infection). The probability of transmission can then be estimated using a binomial distribution,

$$\phi =1-\prod_{g=1..q}^{}\left(1-\left[b_{S\left(g\right)}\left(1-\nu \right)+b_{S\left(g\right)}\nu \;\left(1-\varepsilon_{S\left(g\right)}\right)\right]\right),$$ (1)

where *q* is the number of bites received and *S*(*g*) signifies the residual-sporozoite score in the *g*^th^ bite (i.e. *S*(*g*) = 0,1,2,3 or 4). Parameter *v* is a binary variable denoting whether the vertebrate host received a PEV or not whilst *ε*_*j*_ indicates the efficacy of that PEV against a bite with sporozoite score *j* (the proportional reduction in the probability of infection of a single bite caused by the PEV). Let *i* denote a group of hosts which receive the same parasite challenge (i.e. the same number of bites with the same residual-sporozoite scores and either a PEV vaccine or not), *n*_*i*_ the number of hosts which receive exposure *i* and *x*_*i*_ the number of these that become infected. Using the notation $\underline{x}$ to denote the corresponding list of observations, under a binomial model the likelihood is proportional to,

$$L(\underline{x},\underline{n}|b_{0},b_{1},b_{2},b_{3},b_{4},\nu )\propto {\prod_{i=1..z}\phi^{x_{i}}(1-\phi )}^{n_{i}-x_{i}},$$ (2)

where *z* is the number of combinations of different parasite exposures. This likelihood can be maximised to obtain estimates of the transmission probabilities of mosquitoes with different residual-sporozoite scores from the infection data.

A suite of nested models were fit to the human and mouse data and the most parsimonious were selected using the Akaike information criterion (AIC, lowest value giving the most parsimonious model). These nested models ranged from the full model outlined above (model 6 in S3 Table where mosquitoes with each of the different residual-sporozoite scores has a different contribution to transmission) down to models where all sporozoites positive scores were pooled together (i.e. transmission was independent of sporozoite load but dependent on the number of mosquito bites, model 2) or infection is independent of the number of bites (model 1). Residual-sporozoite scores of zero were also included to determine whether they had an impact on transmission. To test whether a bite with a certain score (or a range of scores) had a significant impact on the transmission metrics each model was systematically reduced to determine whether setting the contribution of a bite to zero improved the parsimony of the model. The full range of different models investigated is outlined in S3 Table. The impact of the PEV was subsequently assessed by initially assuming that vaccination had a different impact on the probability of infection of each sporozoite score before the model was systematically collapsed, grouping the impact of the vaccine on adjacent sporozoite scores together, until the most parsimonious combination was found. Multivariate 95% confidence intervals for each of the parameters were generated from the likelihood profile using a likelihood ratio test. Residual-sporozoite scores (or groups of) whose 95% confidence intervals spanned 0 were further collapsed to determine whether setting them to zero improved the parsimony of the model. To investigate the full impact of (apparently) uninfected bites the datasets were reduced, removing all hosts that only received bites with a residual-sporozoite score of zero. The best fit model was used to predict the likelihood of infection for each experimental infection according to the number of bites they received, the number of residual-sporozoites within those bites and whether or not they received a vaccine candidate. The association between these predicted values and the observed infectious status was evaluated using simple logistic regression (with null and full models compared using a likelihood ratio test).

### Statistical Analysis–Time to Patency

The pre-patent period is typically defined as the first time-point at which infected red blood cells are detected. In this study it is estimated in humans using qPCR [45, 46] and microscopy in mice [24]. The appearance of the first detectable parasite is relatively variable as some PCR runs generate false positive (low) values [47] and the number of parasites in the sample will vary by chance at low densities [48]. This is shown in the dataset by high heteroscedacity when linear random effects models are fit to the individual parasite growth rate curves [47]. Instead studies measure a time to a low parasite density threshold as this reduces sampling variability and the influence of false positive results. Here a density of 1000 parasites per μl for humans and 2% infected red blood cells for mice are used though the same qualitative results are seen with different thresholds. Therefore throughout the manuscript the time to patency specifically refers to the time to the threshold parasite density.

The influence of residual-sporozoite score on the time to patency is analysed using a semi-parametric additive hazard model [49] implemented in the “timereg” R package [50]. This type of survival analysis enables the covariates to act additively on the baseline hazard instead of multiplicatively (as done in standard proportional hazard model) allowing the number of bites to act on an absolute scale rather than a relative one [50]. As there is a minimum time between parasite challenge and patency the baseline hazard is allowed to vary over time whilst the covariates (the number of bites with a score of 0,1,2,3 or 4 and whether the host was given a vaccine) are assumed to be time invariant. In the human dataset a variety of different vaccine candidates were tested. For simplicity it is assumed that all vaccines have an equal impact on the time to patency as the number of volunteers for each vaccine type is very small (typically 3–8 volunteers per candidate, S1 Table). Parasite challenges which did not result in infection were removed from the analysis to ensure that the influence of sporozoite number on the time to patency is independent of whether the host became infected. Infected hosts who had not reached the parasite density threshold (42 out of 429 mice) were classified as being censored. The mean time to patency was estimated by integrating over the survivorship function and the association between model derived prediction and the observed time to patency was tested using simple linear regression.

Likelihood estimates are not available for the additive hazard model so the significance of the different covariates (from zero) is estimated by resampling [50]. Without more formal model selection procedure the full model is collapsed (using the model combinations presented in S3 Table) until all covariates are significantly different from zero (*p* values of <0.05 were significant). For example, if the model collapses to model 2 then this suggests that the time to patency is independent of residual-sporozoite score but is significantly associated with the number of mosquito bites. For some mice only a binary outcome for infection was available instead of a parasite density estimate. To enable the whole dataset to be utilized a dummy variable was added denoting whether parasite density estimates were measured or not, providing an estimate of the time between the first detection and 2% red blood cell infection. Point estimates of the 95% confidence intervals were generated by resampling [50]. Raw data used in the mouse work is given in S1 Dataset.

The size of the Liver-to-Blood parasite Inoculum (LBI) can be estimated using linear regression from the parasite growth rate in the vertebrate host [47]. LBI estimates has been used to disentangle the impact of vaccine in CHMI trials and may be associated with residual-sporozoites number. Unfortunately its estimation is beyond the scope of this work as it requires accurate measures of asexual parasite density over multiple timepoints. This information was also unavailable for the mouse dataset as parasitemia was estimated by microscopy (which has high measurement error, particularly at low densities) and because relatively few data points are available (on average <3 per host above the density threshold). In the human data information from different vaccines is pooled as each study is relatively small. This would make LBI estimates highly uncertain due to differences in the parasite multiplication and variability in PCR heteroscedacity between studies [47].

## Supporting Information

S1 Table Outline of the human and mouse data sets. Values in brackets show the range of the data whilst ▫ denotes studies which received 5 bites from mosquitoes with >100 salivary gland sporozoites post feed. ○ indicates data from this study came from initial challenge and those volunteers which had sterilizing immunity which were re-challenged 6 months later. In the humans all controls patients became infected so could not be used in the probability of infection analysis.
(DOCX)

S2 Table Descriptive statistics of the human and mouse challenge studies. Summary statistics of the residual-sporozoite score of mosquitoes which did or did not infect humans and mice. The right hand column shows the results of linear mixed-models used to determine whether there was a significant difference between infected and uninfected hosts. Values < 0.05 are assumed to be a significant difference.
(DOCX)

S3 Table Outline of the different models investigated and their fit to human and mouse data. The colour of the box indicates whether that residual-sporozoite score (orange = zero, purple = 1–10, green = 11–100, blue = 101–1000, red = >1000) had a distinct probability of infection. For example model 6 is the full model with each sporozoite score having a different probability of infection. The different grey boxes show consecutive residual-sporozoite scores which were grouped together. Model 1 shows the null model where the probability of infection was the same for all mosquitoes irrespective of the number of bites received. Models 2 and 3 shows the scenarios whereby the probability of infection is determined solely by the number of bites or the number of infectious bites, respectively. The models are compared using Akaike information criterion (AIC) with the lower the value the more parsimonious the model (denoted in each column by *). Results are shown for humans with a pre-erythrocytic vaccine candidate (PEV). In mice the datasets with and without PEV are analysed separately and together (assuming resistance reduces the probability of infection equally for bites with different residual-sporozoite scores). In all best fit models mosquitoes with a higher residual-sporozoite score had a higher infection probability.
(DOCX)

S4 Table The semi-parametric components of the best-fit additive hazard models of humans and mice. Additive hazard regression coefficient show the additional hazard in days (see (16) for a full explanation). P-values indicate whether the coefficient is significantly different from zero. In humans overall p-values for the significance of residual sporozoite scores of 2,3 or 4 are not possible as their occurrence is correlated with one another (each volunteer received 5 bites of score 2 or above). However the significance of each of these scores can be seen by comparing Model 10 with Model 13 and 14 (see S3 Table for a list of models). Differentiating between bites of 2 or 3 and 4 (Model 10 vs 14) significantly improves the fit of the model (additive hazard coefficient of 4 = 0.008, p-value = 0.036). Equally differentiating between scores of 2 and 3 or 4 (Model 10 vs 13) showed sporozoite score was also significant here (additive hazard coefficient of 2 = -0.016, p-value = 0.010).
(DOCX)

S1 Fig A diagram illustrating two possible hypotheses for how the efficacy of a vaccine might be associated with the number of residual-salivary gland sporozoites. Panel A repeats Fig 1E from the main text showing how the probability of infection in mice changes with the number of residual-sporozoites (with an arithmetic scale on the x-axis instead of a logarithmic). The number of residual-sporozoites was categorised so the mid-point of each bin is plotted (with the exception of the highest >1000 which takes the value 1200). Colours denote the category of residual-sporozoites, be it 0 (orange), 1–10 (purple), 11–100 (green), 101–1000 (blue) or >1000 (red). Circular points show naïve mice whilst squares show those given the anti-CSP antibody. The non-linear shape of the relationship indicates that the probability of infection is a negative density-dependent process. Panel B uses a made up graph to illustrate how this non-linear relationship can cause the efficacy to change according to the number of residual-sporozoites. The solid black line denotes a hypothetical relationship between the probability of infection and the number of residual sporozoites (similar to that observed in Panel A) whilst the other lines give different hypotheses for the action of a pre-erythrocytic vaccine. The pink dashed line assumes that the vaccine reduces the per-sporozoite transmission probability by 50% (here referred to as a “leaky” dose response) whilst the brown dotted-dashed line assumes that the vaccine reduces the number of sporozoites surviving in the host by a constant amount (the same across all hosts) so no mosquito with <400 residual-sporozoites is capable of transmitting the infection (here referred to as a “threshold” dose response). Panel C shows how these two hypotheses influence the relationship between vaccine efficacy and the number of residual-sporozoites. Further work is required to differentiate between these hypothesis though the human data presented here is consistent with the “threshold” dose response as only bites with >1000 residual-sporozoites appeared to contribute to transmission.
(DOCX)

S1 Dataset Raw mouse data used to fit the probability of infection and time to patency models. (XLSX)
