# Supplementary material for: Probability of Transmission of Malaria from Mosquito to Human Is Regulated by Mosquito Parasite Density in Naïve and Vaccinated Hosts
Source: PLoS Pathog. 2017 Jan 12;13(1):e1006108. doi: 10.1371/journal.ppat.1006108 (PMC5230737; doi:10.1371/journal.ppat.1006108)
Supplement: S1 Table — Values in brackets show the range of the data whilst ▫ denotes studies which received 5 bites from mosquitoes with >100 salivary gland sporozoites post feed. ○ indicates data from this study came from initial challenge and those volunteers which had sterilizing immunity which were re-challenged 6 months later. In the humans all controls patients became infected so could not be used in the probability of infection analysis. (DOCX) [file ppat.1006108.s001.docx]

| **Reference and detail** | | | | | **Number of hosts with PEV** | **Number of naïve hosts** |
| --- | --- | --- | --- | --- | --- | --- |
| **Humans** | | | | |  |  |
| *Probability of infection* | | | | |  |  |
|  | ·RTS,S/AS01_B_, with or without simian adenovirus 63 (ChAd63) and modified vaccinia virus Ankara (MVA) expressing the multiple epitope–thrombospondin-related adhesion protein (ME-TRAP) [1] | | | | 47^○^ | 0 |
|  | | | | |  |  |
| *Time to patency* | | | | |  |  |
|  | ·MVA ME-TRAP, with or without prior DNA ME-TRAP [2]▫ | | | | 16 | 4 |
|  | ·Recombinant fowlpox virus (FP9), DNA/MVA ME-TRAP [3]▫ | | | | 7 | 3 |
|  | RTS,S/AS02A and MVA CSP [4]▫ | | | | 8 | 5 |
|  | ·DNA and MVA expressing ME-TRAP and CSP [5]▫ | | | | 15 | 6 |
|  | ·Modified hepatitis B core protein with B cell and CSP epitopes [6] | | | | 13 | 6 |
|  | ·FP9 and MVA encoding CSP and ME-TRAP in different combinations, doses and application [7] | | | | 15 | 5 |
|  | ·FP9 and MVA expressing a 3240 amino-acid long polyprotein (FP9-PP and MVA-PP) [8] | | | | 15 | 6 |
|  | ·PEV3A, expressing CSP and Apical membrane antigen 1 with or without FP9/MVA ME-TRAP [9] | | | | 22 | 6 |
|  | ·Different regimen of ChAd63 or MVA ME-TRAP [10] | | | | 42 | 12 |
|  | ·ChAd63-MVA CSP and ChAd63-MVA ME-TRAP [11] | | | | 18 | 6 |
|  | ·DNA and MVA expressing ME-TRAP and CSP (those with blood-stage components, apical membrane antigen-1, excluded) [12] | | | | 11 | 5 |
|  | ·RTS,S/AS01_B_ with or without ChAd63 ME-TRAP and MVA ME-TRAP [13] | | | | 10 | 11 |
|  | **Total** | | | | **192** | **75** |
| **Mice** | |  |  |  |  |  |
| *Probability of infection* | |  |  |  |  |  |
|  | ·Naïve mice [14] | | | | 0 | 245 |
|  | ·Naïve mice [15] | | | | 0 | 320 |
|  | ·With and without anti-CSP antibody 3D11 (unpublished) | | | | 89 | 190 |
|  | **Total** | | | | **89** | **755** |
| *Time to patency* | |  |  |  |  |  |
|  | ·Naïve mice [14] | | | | 0 | 145 |
|  | ·Naïve mice [15] | | | | 0 | 253 |
|  | ·With and without anti-CSP antibody 3D11 (unpublished) | | | | 31 | 0 |
|  | **Total** | | | | **31** | **398** |

**Additional References**

1. Rampling T, Ewer KJ, Bowyer G, Bliss CM, Edwards NJ, Wright D, et al. Safety and High Level Efficacy of the Combination Malaria Vaccine Regimen of RTS,S/AS01B with ChAd-MVA Vectored Vaccines Expressing ME-TRAP. J Infect Dis. 2016. doi: 10.1093/infdis/jiw244. PubMed PMID: 27307573.

2. Moorthy VS, Pinder M, Reece WH, Watkins K, Atabani S, Hannan C, et al. Safety and immunogenicity of DNA/modified vaccinia virus ankara malaria vaccination in African adults. J Infect Dis. 2003;188(8):1239-44. doi: 10.1086/378515. PubMed PMID: 14551895.

3. Webster DP, Dunachie S, Vuola JM, Berthoud T, Keating S, Laidlaw SM, et al. Enhanced T cell-mediated protection against malaria in human challenges by using the recombinant poxviruses FP9 and modified vaccinia virus Ankara. Proc Natl Acad Sci U S A. 2005;102(13):4836-41. doi: 10.1073/pnas.0406381102. PubMed PMID: 15781866; PubMed Central PMCID: PMC555695.

4. Dunachie SJ, Walther M, Vuola JM, Webster DP, Keating SM, Berthoud T, et al. A clinical trial of prime-boost immunisation with the candidate malaria vaccines RTS,S/AS02A and MVA-CS. Vaccine. 2006;24(15):2850-9. doi: 10.1016/j.vaccine.2005.12.041. PubMed PMID: 16434127.

5. Dunachie SJ, Walther M, Epstein JE, Keating S, Berthoud T, Andrews L, et al. A DNA prime-modified vaccinia virus ankara boost vaccine encoding thrombospondin-related adhesion protein but not circumsporozoite protein partially protects healthy malaria-naive adults against *Plasmodium falciparum* sporozoite challenge. Infect Immun. 2006;74(10):5933-42. doi: 10.1128/IAI.00590-06. PubMed PMID: 16988273; PubMed Central PMCID: PMC1594937.

6. Walther M, Dunachie S, Keating S, Vuola JM, Berthoud T, Schmidt A, et al. Safety, immunogenicity and efficacy of a pre-erythrocytic malaria candidate vaccine, ICC-1132 formulated in Seppic ISA 720. Vaccine. 2005;23(7):857-64. doi: 10.1016/j.vaccine.2004.08.020. PubMed PMID: 15603885.

7. Walther M, Thompson FM, Dunachie S, Keating S, Todryk S, Berthoud T, et al. Safety, immunogenicity, and efficacy of prime-boost immunization with recombinant poxvirus FP9 and modified vaccinia virus Ankara encoding the full-length *Plasmodium falciparum* circumsporozoite protein. Infect Immun. 2006;74(5):2706-16. doi: 10.1128/IAI.74.5.2706-2716.2006. PubMed PMID: 16622207; PubMed Central PMCID: PMC1459746.

8. Porter DW, Thompson FM, Berthoud TK, Hutchings CL, Andrews L, Biswas S, et al. A human Phase I/IIa malaria challenge trial of a polyprotein malaria vaccine. Vaccine. 2011;29(43):7514-22. doi: 10.1016/j.vaccine.2011.03.083. PubMed PMID: 21501642; PubMed Central PMCID: PMC3195259.

9. Thompson FM, Porter DW, Okitsu SL, Westerfeld N, Vogel D, Todryk S, et al. Evidence of blood stage efficacy with a virosomal malaria vaccine in a phase IIa clinical trial. PLoS One. 2008;3(1):e1493. doi: 10.1371/journal.pone.0001493. PubMed PMID: 18231580; PubMed Central PMCID: PMC2204057.

10. Ewer KJ, O'Hara GA, Duncan CJ, Collins KA, Sheehy SH, Reyes-Sandoval A, et al. Protective CD8+ T-cell immunity to human malaria induced by chimpanzee adenovirus-MVA immunisation. Nat Commun. 2013;4:2836. doi: 10.1038/ncomms3836. PubMed PMID: 24284865; PubMed Central PMCID: PMC3868203.

11. Hodgson SH, Ewer KJ, Bliss CM, Edwards NJ, Rampling T, Anagnostou NA, et al. Evaluation of the efficacy of ChAd63-MVA vectored vaccines expressing circumsporozoite protein and ME-TRAP against controlled human malaria infection in malaria-naive individuals. J Infect Dis. 2015;211(7):1076-86. doi: 10.1093/infdis/jiu579. PubMed PMID: 25336730; PubMed Central PMCID: PMC4354983.

13. Rampling T, Ewer KJ, Bowyer G, Bliss CM, Edwards NJ, Wright D, et al. Safety and High Level Efficacy of the Combination Malaria Vaccine Regimen of RTS,S/AS01B with ChAd-MVA Vectored Vaccines Expressing ME-TRAP. J Infect Dis. 2016. doi: 10.1093/infdis/jiw244. PubMed PMID: 27307573.

14. Blagborough AM, Churcher TS, Upton LM, Ghani AC, Gething PW, Sinden RE. Transmission-blocking interventions eliminate malaria from laboratory populations. Nat Commun. 2013;4:1812. doi: 10.1038/ncomms2840. PubMed PMID: 23652000.

15. Upton LM, Brock PM, Churcher TS, Ghani AC, Gething PW, Delves MJ, et al. Lead clinical and preclinical antimalarial drugs can significantly reduce sporozoite transmission to vertebrate populations. Antimicrob Agents Chemother. 2015;59(1):490-7. doi: 10.1128/AAC.03942-14. PubMed PMID: 25385107; PubMed Central PMCID: PMC4291391.
