# Supplementary material for: Probability of Transmission of Malaria from Mosquito to Human Is Regulated by Mosquito Parasite Density in Naïve and Vaccinated Hosts
Source: PLoS Pathog. 2017 Jan 12;13(1):e1006108. doi: 10.1371/journal.ppat.1006108 (PMC5230737; doi:10.1371/journal.ppat.1006108)
Supplement: S2 Table — Summary statistics of the residual-sporozoite score of mosquitoes which did or did not infect humans and mice. The right hand column shows the results of linear mixed-models used to determine whether there was a significant difference between infected and uninfected hosts. Values < 0.05 are assumed to be a significant difference. (DOCX) [file ppat.1006108.s002.docx]

| *Humans given a pre-erythrocytic vaccine* | | Mean residual-sporozoite score in mosquito bites | | |
| --- | --- | --- | --- | --- |
|  |  | Uninfected host | Infected host | *p* value |
|  | Number of hosts | 38 | 9 | - |
|  | Number of bites (range) | 7.05 (5,12) | 7.00 (5,10) | - |
|  | Mean residual-sporozoite score per bite (CI) | 3.35 (3.15,3.53) | 3.36 (3.02,3.72) | = 0.937 |
|  | Total residual-sporozoite score (CI) | 23.5 (21.3,25.9) | 23.4 (19.8,27.6) | = 0.836 |
|  | |  |  |  |
| *Mice given anti-CSP antibodies* | |  |  |  |
|  | Number of hosts | 57 | 32 | - |
|  | Number of bites (range) | 2.37 (1,10) | 6.84 (1,10) | - |
|  | Mean residual-sporozoite score per bite (CI) | 0.23 (0.11,0.36) | 1.81 (1.53,2.07) | <0.0001 |
|  | Total residual-sporozoite score (CI) | 1.02 (0.42,1.76) | 12.0 (9.58,14.9) | <0.0001 |
|  | |  |  |  |
| *Naïve Mice* | |  |  |  |
|  | Number of hosts | 367 | 388 | - |
|  | Number of bites (range) | 3.88 (1,10) | 5.06 (1,10) | - |
|  | Mean residual-sporozoite score per bite (CI) | 0.24 (0.20, 0.30) | 1.36 (1.28,1.44) | <0.0001 |
|  | Total residual-sporozoite score (CI) | 0.93 (0.75,1.13) | 6.26 (5.80,6.70) | <0.0001 |
