# Supplementary material for: Probability of Transmission of Malaria from Mosquito to Human Is Regulated by Mosquito Parasite Density in Naïve and Vaccinated Hosts
Source: PLoS Pathog. 2017 Jan 12;13(1):e1006108. doi: 10.1371/journal.ppat.1006108 (PMC5230737; doi:10.1371/journal.ppat.1006108)
Supplement: S3 Table — The colour of the box indicates whether that residual-sporozoite score (orange = zero, purple = 1–10, green = 11–100, blue = 101–1000, red = >1000) had a distinct probability of infection. For example model 6 is the full model with each sporozoite score having a different probability of infection. The different grey boxes show consecutive residual-sporozoite scores which were grouped together. Model 1 shows the null model where the probability of infection was the same for all mosquitoes irrespective of the number of bites received. Models 2 and 3 shows the scenarios whereby the probability of infection is determined solely by the number of bites or the number of infectious bites, respectively. The models are compared using Akaike information criterion (AIC) with the lower the value the more parsimonious the model (denoted in each column by *). Results are shown for humans with a pre-erythrocytic vaccine candidate (PEV). In mice the datasets with and without PEV are analysed separately and together (assuming resistance reduces the probability of infection equally for bites with different residual-sporozoite scores). In all best fit models mosquitoes with a higher residual-sporozoite score had a higher infection probability. (DOCX) [file ppat.1006108.s003.docx]

| Model no. | Residual-sporozoite number range | | | | | No. para-meters | **AIC** | | | |
| --- | --- | --- | --- | --- | --- | --- | --- | --- | --- | --- |
|  | Zero | 1-10 | 11-100 | 101-1000 | >1000 |  | **Humans**^‡^ | **Mice** | | |
|  |  |  |  |  |  |  | **with PEV** | **without PEV** | **with PEV** | **all data** **^§^** |
| 1 | No information on mosquito biting | | | | | 1 | 47.7 | 1048.1 | 125.5 | 1166.3 |
| 2 |  |  |  |  |  | 1 | 47.7 | 1062.2 | 79.6 | 1141.9 |
| 3 |  |  |  |  |  | 2 | 47.9^†^ | 601.2 | 50.7^†^ | 652.9 |
| 4 |  |  |  |  |  | 3 | 47.9^†^ | 579.8 | 48.6^†^ | 628.2 |
| 5 |  |  |  |  |  | 4 | 49.4^†^ | 577.0 | 48.1^†^ | 624.1 |
| 6 |  |  |  |  |  | 5 | 52.3^†^ | **576.6*** | 49.4^†^ | **623.5*** |
| 7 |  |  |  |  |  | 4 | 49.3^†^ | 655.7 | 50.8 | 703.0 |
| 8 |  |  |  |  |  | 3 | 44.3^†^ | 816.1 | 52.4 | 868.1 |
| 9 |  |  |  |  |  | 2 | **42.3^†^*** | 949.6 | 67.9 | 1016.4 |
| 10 |  |  |  |  |  | 2 | 47.9^†^ | 658.1 | 49.9 | 715.3 |
| 11 |  |  |  |  |  | 2 | 47.4^†^ | 816.7 | 51.5 | 869.2 |
| 12 |  |  |  |  |  | 3 | 49.4^†^ | 656.0 | 49.5 | 703.6 |
| 13 |  |  |  |  |  | 3 | 49.4^†^ | 656.0 | 49.5 | 703.6 |
| 14 |  |  |  |  |  | 3 | 44.3^†^ | 655.0 | 50.7 | 702.9 |
| 15 |  |  |  |  |  | 3 | 44.3^†^ | 590.6 | 50.0 | 640.4 |
| 16 |  |  |  |  |  | 4 | 46.3^†^ | 579.3 | 49.4^†^ | 625.8 |
| 17 |  |  |  |  |  | 4 | 46.3^†^ | 583.3 | **47.7^†^*** | 630.9 |

^†^ Setting the lowest residual-sporozoite score (or group of scores) to zero reduced the AIC

^‡^ For the human dataset including information on the number of “unfed” mosquitoes did not reduce the AIC. These data was not available for the murine system.

^§^ Dataset with and without PEV using equations [1] and [2] used in Figure 1E of main text
