# Supplementary material for: Probability of Transmission of Malaria from Mosquito to Human Is Regulated by Mosquito Parasite Density in Naïve and Vaccinated Hosts
Source: PLoS Pathog. 2017 Jan 12;13(1):e1006108. doi: 10.1371/journal.ppat.1006108 (PMC5230737; doi:10.1371/journal.ppat.1006108)
Supplement: S4 Table — Additive hazard regression coefficient show the additional hazard in days (see (16) for a full explanation). P-values indicate whether the coefficient is significantly different from zero. In humans overall p-values for the significance of residual sporozoite scores of 2,3 or 4 are not possible as their occurrence is correlated with one another (each volunteer received 5 bites of score 2 or above). However the significance of each of these scores can be seen by comparing Model 10 with Model 13 and 14 (see S3 Table for a list of models). Differentiating between bites of 2 or 3 and 4 (Model 10 vs 14) significantly improves the fit of the model (additive hazard coefficient of 4 = 0.008, p-value = 0.036). Equally differentiating between scores of 2 and 3 or 4 (Model 10 vs 13) showed sporozoite score was also significant here (additive hazard coefficient of 2 = -0.016, p-value = 0.010). (DOCX) [file ppat.1006108.s004.docx]

| **Variable** | **Humans** | | **Mice** | |
| --- | --- | --- | --- | --- |
|  | **Coefficient**  **(standard error)** | ***p ­*– value** | **Coefficient**  **(standard error)** | ***p ­*– value** |
| **Score of 0&1** | 0.009 (0.003) | <0.001 | 0.007 (0.002) | <0.001 |
| **Score of 2** | -0.013 (0.01) | - | 0.018 (0.005) | <0.001 |
| **Score of 3** | -0.000 (0.01) | - | 0.037 (0.006) | <0.001 |
| **Score of 4** | 0.005 (0.01) | - | 0.044 (0.006) | <0.001 |
| **PEV** **^¶^** | -0.021 (0.013) | 0.035 | 0.171 (0.013) | <0.001 |

**^¶^** Binary variable denoting whether vertebrate was given a pre-erythrocytic vaccine or antibody
