# Supplementary material for: Probability of Transmission of Malaria from Mosquito to Human Is Regulated by Mosquito Parasite Density in Naïve and Vaccinated Hosts
Source: PLoS Pathog. 2017 Jan 12;13(1):e1006108. doi: 10.1371/journal.ppat.1006108 (PMC5230737; doi:10.1371/journal.ppat.1006108)
Supplement: S1 Fig — Panel A repeats Fig 1E from the main text showing how the probability of infection in mice changes with the number of residual-sporozoites (with an arithmetic scale on the x-axis instead of a logarithmic). The number of residual-sporozoites was categorised so the mid-point of each bin is plotted (with the exception of the highest >1000 which takes the value 1200). Colours denote the category of residual-sporozoites, be it 0 (orange), 1–10 (purple), 11–100 (green), 101–1000 (blue) or >1000 (red). Circular points show naïve mice whilst squares show those given the anti-CSP antibody. The non-linear shape of the relationship indicates that the probability of infection is a negative density-dependent process. Panel B uses a made up graph to illustrate how this non-linear relationship can cause the efficacy to change according to the number of residual-sporozoites. The solid black line denotes a hypothetical relationship between the probability of infection and the number of residual sporozoites (similar to that observed in Panel A) whilst the other lines give different hypotheses for the action of a pre-erythrocytic vaccine. The pink dashed line assumes that the vaccine reduces the per-sporozoite transmission probability by 50% (here referred to as a “leaky” dose response) whilst the brown dotted-dashed line assumes that the vaccine reduces the number of sporozoites surviving in the host by a constant amount (the same across all hosts) so no mosquito with <400 residual-sporozoites is capable of transmitting the infection (here referred to as a “threshold” dose response). Panel C shows how these two hypotheses influence the relationship between vaccine efficacy and the number of residual-sporozoites. Further work is required to differentiate between these hypothesis though the human data presented here is consistent with the “threshold” dose response as only bites with >1000 residual-sporozoites appeared to contribute to transmission. (DOCX) [file ppat.1006108.s005.docx]

C

B

A
